# Advancing mobile learning in Australian healthcare environments: nursing profession organisation perspectives and leadership challenges

**DOI:** 10.1186/s12912-018-0313-z

**Published:** 2018-11-12

**Authors:** Carey Ann Mather, Elizabeth Anne Cummings, Fred Gale

**Affiliations:** 1 School of Health Sciences, College of Health and Medicine, University of Tasmania, Locked Bag 1322, Launceston, TAS 7250 Australia; 2 School of Health Sciences, College of Health and Medicine, University of Tasmania, Private Bag 135, Hobart, TAS 7001 Australia; 3 School of Social Sciences, College of Arts, Law and Education, University of Tasmania, Locked Bag 1340, Launceston, TAS 7250 Australia

**Keywords:** Agency, Continuing professional development, Digital professionalism, Governance, Mobile learning, Mobile technology, Nursing, Point of care

## Abstract

**Background:**

Access to, and use of, mobile or portable devices for learning at point of care within Australian healthcare environments is poorly governed. An absence of clear direction at systems, organisation and individual levels has created a mobile learning paradox, whereby although nurses understand the benefits of seeking and retrieving discipline or patient-related knowledge and information in real-time, mobile learning is not an explicitly sanctioned nursing activity. The purpose of this study was to understand the factors influencing mobile learning policy development from the perspective of professional nursing organisations.

**Methods:**

Individual semi-structured interviews were undertaken with representatives from professional nursing organisations in December 2016 and January 2017. Recruitment was by email and telephone. Qualitative analysis was conducted to identify the key themes latent in the transcribed data.

**Results:**

Risk management, perceived use of mobile technology, connectivity to information and real-time access were key themes that emerged from the analysis, collectively identifying the complexity of innovating within an established paradigm. Despite understanding the benefits and risks associated with using mobile technology at point of care, nursing representatives were reluctant to exert agency and challenge traditional work patterns to alter the status quo.

**Conclusions:**

The themes highlighted the complexity of accessing and using mobile technology for informal learning and continuing professional development. Mobile learning cannot occur at point of care until the factors identified are addressed. Additionally, a reluctance by nurses within professional organisations to advance protocols to govern digital professionalism needs to be overcome. For mobile learning to be perceived as a legitimate nursing function requires a more wholistic approach to risk management that includes all stakeholders, at all levels. The goal should be to develop revised protocols that establish a better balance between the costs and benefits of access to information technology in real-time by nurses.

## Background

The use of mobile technology to access information in real-time is ubiquitous in modern life. Digital knowledge transfer is an outcome of using mobile technology that is currently underutilised in Australian healthcare settings [1]. Harnessing mobile learning to augment traditional andragogies in healthcare environments by stakeholders, especially nurses, has been slow. Previous studies to explore the lack of mobile learning at point of care by nurses have been undertaken [1–4]. Focus group studies with nurse supervisors and online surveys with students have uncovered barriers, challenges, risks and benefits to nurses and undergraduate students of being able to access and use mobile technology for learning at point of care [3–5]. Analysis of the Registered Nurse Standards for Practice [6] and professional Codes of Conduct [7, 8] have revealed an absence of guidance to support this adjunct method of learning.

The aim of this study was to explore the factors influencing the governance of mobile technology at point of care for informal learning and continuing professional development (CPD) from the perspectives of representatives of professional nursing organisations. Barriers, risks, challenges and benefits to using mobile technology by nurses at point of care have been previously been identified in the international literature at individual, organisational and systems levels [9–12]. Inadequate governance and lack of understanding within registered health professions regarding the potential of accessing and using mobile technology for learning has created further disruption to healthcare provision both within Australia and internationally [13–15]. The resultant inability of nurses to use mobile technology for informal learning and CPD at point of care in Australia hinders them meeting the annual learning requirements for registration as a nurse [16, 17]. Additionally, the lack of legitimate access to mobile learning prohibits nurses from guiding and supporting student nurses and modelling digital professionalism, while undertaking work integrated learning within healthcare environments.

Clear direction regarding governance of mobile technology for leisure and learning within healthcare settings remains unaddressed at a systems level in nursing, with flow-on effects impacting at the organisation and individual levels. While nursing informatics is now an essential component of the undergraduate nursing curriculum [18, 19], students and registered nurses are not formally or consistently taught digital professionalism. In the resulting confusion regarding appropriate and safe use of mobile technology at point of care, opportunities arise for advertent and inadvertent professional transgressions to occur [20]. The blurring of public-private boundaries in healthcare environments generates organisational risks and potential adverse media attention if nurses make poor choices regarding access and use of mobile technology. Fear of litigation has negatively impacted the ability of nurses to access mobile technology in the workplace as organisations have dissuaded its use. Paradoxically, however, nursing is consistently reported to be the most trustworthy profession [21], with nurses depended on to provide complex nursing care and administer controlled substances, yet not trusted with carrying a mobile device to access information at point of care [2, 22].

Nurses are the largest group within the registered health professions in Australia [23], making it costly for organisations to educationally prepare their nursing workforce to become proficient in using mobile technology at point of care. However, a digitally capable workforce will be also be able guide the new generation of nurses to become digitally professional and minimise the potential risks associated with using digital media. Upskilling the nursing workforce will also contribute to lessening the current confusion whereby undergraduate students can use mobile technology for learning [11, 24] except during work integrated learning [25]. Promotion of congruency in mobile learning opportunities across the profession is now necessary if nursing is to remain contemporary and continue to be viewed by the public as a trustworthy profession [21, 26].

Research on generational cohorts has previously focused on the retiring ‘Baby Boomers’ (1946–1964) who were vested with the responsibility of initiating digital technology into occupations [27] and ‘Generation Y’ (1982–1995) who have grown up with access to digital technology and are currently entering the workforce [28, 29]. However, there is now research indicating ‘Generation X’ (those born between 1965 and 1982) are hindering the installation of mobile technology into healthcare environments. Christopher and colleagues [30] report Generation X nurses in senior management positions believe they have insufficient formal powers to be innovative within healthcare environments. This lack of influence may manifest as an inability to promote mobile learning as a legitimate nursing function [31]. Research about ‘Generation Y’ indicates a dislike of hierarchy [29, 32]. This aversion exhibits as Generation Y being reluctant to challenge existing work structures, including those activities that could promote a ‘learning organisation’ [30, 33] This generational dissonance regarding structural empowerment may contribute to the lack of agency demonstrated by nurses to lead implementation of mobile learning as a legitimate nursing function.

Mobile technology enables individuals to seek and retrieve information in real-time that can aid in decision-making that could potentially improve patient outcomes [34, 35]. Access to information at point of care also has the potential to improve workflow. Westbrook and colleagues [36] quantified patterns of task time distribution and found nurses completed an average of 72.3 tasks per hour which over time became more fragmented and interrupted, creating potential safety concerns. Deployment of mobile learning has the potential to reduce this fragmentation by enabling continuity of care of patients, as nurses would not need to leave the bedside to check or clarify information. This study targeted representatives from nursing profession organisations to better understand from their perspective the factors influencing the use of mobile technology for informal learning and CPD.

## Methods

### Design

This research uses interpretive description as discussed by Thorne [37]. It draws on the work of Creswell [38], and Strauss and Corbin [39] by using purposive sampling and employing a reflexive approach within a systematic framework to code, label and categorise the data to enable analysis.

### Participants and recruitment

Purposive sampling was used to recruit participants from a range of nursing profession organisations. Inclusion criteria for interview were being a nurse employed or belonging to a nursing profession organisation senior enough to be able to represent the organisation from a policy or guideline perspective and having expertise in nursing practice. A potential list of organisations was generated (CM and EC) that included National (*n* = 7) and Coalition of National Nursing and Midwifery Organisations (CoNNMO) member (*n* = 55) organisations. Invitations were sent to the contact emails provided via the national organisation or CoNNMO website (*n* = 52). If no response was received within two weeks, a follow-up telephone call was made. If there were no telephone contact details available, a further email was sent to the same address. A reminder email was despatched one month after the initial email invitation. An information sheet was provided as an attachment to the email invitation and consent to participate was recorded prior to the beginning of the recorded interview as per ethics protocol for approval H0016097.

### Data collection

Interviews with participants were conducted and recorded using Skype for Business™, at a mutually agreeable time using a semi-structured schedule as a guide. The interview schedule question development was informed by previous research [4] and developed by two researchers (CM and EC). Prompts and potential probing questions were included in the schedule to maintain congruency of questioning (Table 1). Interview questions were designed to establish whether the nursing profession organisations had a policy position on mobile technology for informal learning and CPD and then to explore factors impacting the use of mobile technology for learning at point of care.

**Table 1:** Nursing profession representative interview schedule

Number	Question
1	Can you tell me about the overall view of this organisation’s position on nurses and midwives using mobile technology for informal learning and CPD in the workplace?
2	If your organisation has a position on mobile technology use for mobile learning, please provide detail about how this position was developed?
3	If your organization has no position on mobile technology use for mobile learning, what do you think this organisation could offer in order to influence the use of mobile technology for informal learning and CPD in the workplace?
4	Can you tell me what your organisation can do to support the development of standards, guidelines or policies about the access and use of mobile technology at point of care?
5	Can you explain to me in your own words how portable or mobile technology could change learning in the workplace?
6	Can you tell me about how your organisation’s opinion on access to portable or mobile learning environments impact on patient or client safety?
7	Can you tell me about your organisation’s opinion on perceptions of public about nurses and/or midwives using portable or mobile technology in the workplace?
8	Do you have any opinion on perceptions of other health professionals using portable or mobile technology in the workplace?
9	What do you perceive nurses or midwives currently do for continuing professional development to meet the requirements for AHPRA?
10	Do you have any other comments you would like to make regarding nurses using mobile technology for learning?

The interviews were conducted during December 2016 and January 2017, took between 17.29 and 54.29 min (mean 34.05 min) and were transcribed verbatim. Variations in interview length were due to the depth of knowledge of the topic of investigation by individual participants.

### Data analysis

A systematic and organised process was developed consisting of trial coding with member checking and development of a codebook that provided a framework of codes. Auditing of codes and reviewing previous interviews to ensure consistency of application of labels across interviews was conducted during the process of coding. Inductive thematic analysis was undertaken by coding ‘meaning units’ as ‘open codes’ as described by Elliot and Timulak [40]. ‘Meaning units’ were tabulated in Microsoft Excel (2016), from which data was labelled and reduced from open to axial and finally to selective codes to enable the sub-themes to be revealed. This process of labelling and reducing the phrases by coding enabled further refinement of the data to become four core themes. Constant comparison was undertaken by two of the authors (CM and EC).

### Rigour

The interviewer (CM) familiarised herself with the schedule to ensure the interview process flowed and enabled probing questions and prompts to be less rehearsed. The interviewer was aware of the lack of body language cues and maintained a neutral but encouraging dialogue with participants [41]. At the conclusion of each interview, interviewees were asked if they had any further information they would like to add. This opportunity enabled participants to raise any issues or information that had not been discussed during the interview. The accuracy of the transcriptions were confirmed, by reading and listening to the audio recordings of the interviews simultaneously by the interviewer. At the conclusion of each interview, participants were offered the opportunity to check the transcription for errors. This process minimised potential for error and ensured accuracy of the data transcription.

### Ethics

Ethics approval was gained from The University of Tasmania Social Sciences Human Research Ethics Committee (H0016097) prior to commencement of the study as required under Australia’s National Statement on Ethical Conduct in Human Research [42].

## Results

### Participant demographics

Six interviews were conducted during the study period (Table 2) Participants were senior registered nurses holding executive positions who through their careers had a broad range of nursing experiences in a variety of healthcare settings. They were paid employees or were volunteers within Australian nursing specialty organisations that were members of CoNNMO.

**Table 2:** Participant demographics

Interview	Nursing organisation	Nurse role	Source of recruitment	Gender
1	National representative (Executive)	Administration	Direct email to organisation	F
2	Specialty nursing Executive position (volunteer organisation)	University academic and clinician	Via email from CoNNMO secretariat^a^	M
3	National representative (Executive)	University academic	Direct email to organisation	F
4	Specialty nursing Executive position (volunteer organisation)	Clinician	Via email from CoNNMO secretariat	F
5	Specialty nursing Executive position (volunteer organisation)	Administration and clinician	Via email from CoNNMO secretariat	M
6	National representative (Executive)	Administration	Direct email	F

CoNNMO^a^ Coalition of National Nursing and Midwifery Organisations

Gaining access to appropriate nursing representatives to seek participation proved problematic owing to the complexity of the national organisations targeted or voluntary nature of the membership to nursing specialty organisations. The lead time required to obtain national organisation permission to interview varied. Requests for interviewing a representative from the organisation needed to be taken to appropriate internal meetings to be considered. Feedback from organisations was sought, after meetings were held to discuss the interview request. However, reaching an appropriate representative for interview remained complicated. One organisation declined to participate due to a decision made by the organisation Director. Access to nursing speciality organisation representatives affiliated with CoNNMO was ad hoc, owing to the volunteer nature of many nursing specialty organisations. The voluntary nature of these organisations was apparent by irregular monitoring of email accounts, so non-acknowledgement or response from point of entry was common. However, initial and follow-up contact was undertaken as per ethics protocol. The complexity of gaining access to National representatives and the poor response from voluntary organisations impacted the capacity to recruit interviewees. In addition, the release of the Australian College of Nursing, Health Informatics Society of Australia and Nursing Informatics Australia joint draft position statement on health informatics in February 2017 resulted in cessation of recruitment as the researchers believed it could influence the responses of future participants.

### Themes

Four key themes emerged from the data analysis, revealing the complexity of factors that influence governance of mobile learning at point of care in healthcare environments in Australia. These themes were: 1) risk management; 2) perceived use of mobile technology; 3) connectivity to information; and 4) real-time access. Addressing all four themes was found to be imperative for enabling mobile learning at point of care.

### Risk management

Participants identified numerous potential risks in employing mobile technology at point of care that required management to minimise adverse or unintended consequences. Participants acknowledged there was a lack of governance at a wider systems level that negatively impacted their capacity to use mobile technology at point of care. They indicated the belief that mobile technology was not allowed within healthcare settings. The belief was expressed that the non-use of mobile technology had developed historically, with one participant stating:

*“We also had, I think we've still got some of the misconceptions around the risks with mobile devices and medical devices” (Participant 2).*

Another influence on the lack of direction regarding mobile learning within organisations was attributed to generational cohorts. One participant reported:

*“But we have to overcome the establishment, the bureaucracy in the health system that actually sees this as a bad thing, that oh no, they’re going to be on social media and they’re all going to be doing bad things and this instant thought that the internet is just this bad place and no good will come of it. I think some of the older directors of nursing and all that sort of stuff, who are all basically starting to retire now sort of are making way for a younger generation of directors of nursing who we hope is going to have a better or a more positive approach to this” (Participant 5).*

Representatives described factors that have influenced organisations to implement policies or local rules within organisations excluding the use of mobile technology. Participants cited organisations formally and informally dissuading nurses from using mobile technology at point of care. This was expressed by one representative who stated:

*“But the nurses I find, whether it’s just that they’re more regulated, are not encouraged to use their phones in the actual clinical environment” (Participant 4).*

Interviewees reported there was inconsistency of access and use, which created confusion for nurses within organisations as shown by this comment:

*“But unfortunately, it’s such a reactive approach rather than proactive approach, in that they’re not - it’s actually,” “Well, the technology’s great, most people are using it appropriately, but you can’t stop every - you know, don’t stop everybody from using it because some people have been not doing the right thing” (Participant 3).*

They acknowledged incongruency with using mobile technology for patient care, clinical decision-making and the lack of capacity for seeking and retrieving information at point of care. Nurses expressed concern over the lack of direction provided to the profession at a National level, which then impacted at an organisation level. Participants provided examples of other health professionals’ expectations of nurses being able to access mobile technology even when organisational policy precluded its use. One participant stated:

*“But then, as I said, there’s that conflict between, we’re encouraged to have those things on our phone, but we’re not allowed to really use them on the ward. So, there is an issue around that, that you will send a photo to a consultant and actually, that is written into policy that that’s a breach of that particular policy; you’re not allowed to send patient’s photos on personal devices” (Participant 4).*

Interviewees revealed that although there was little formal direction at a systems level on whether mobile technology could be used, some nursing staff were beginning to challenge the apparent edict to drive change:

*“What they have now, so we're living in a bit of a fantasy world at the moment where people say there's no mobile phones allowed, when in fact everyone has a mobile phone in their pocket” (Participant 2).*

A participant indicated another influence on practice was previous breaches of patient confidentiality or privacy, which motivated organisations to limit access to mobile technology:

*“I think it will be when - we’ve had - the reason it’s come about unfortunately, is because of the opposite reason, in that people’s photos have got out onto Facebook and to general internet public forums and there’s been people that have been sued” (Participant 4).*

Cyberloafing behaviour was cited as a reason for preventing legitimate access to mobile technology while at work. One representative expressed:

*“And I don’t know whether it’s a different generation or different - that people think that they might be checking Facebook, or they might be misusing their mobile devices rather than using them for education” (Participant 4).*

Participants offered potential workarounds to resolve the current impasse regarding legitimate use of mobile technology at point of care, indicating they believed nurses were capable of discerning when mobile learning could be deployed:

*“We’re good at coming up with solutions to things. And I think that’s part of our learning” (Participant 6).*

One participant summed up the current situation related to guidance of mobile learning at point of care within Australian healthcare environments by stating:

*“So, it is a real messy minefield” (Participant 2).*

Interviewees raised the importance of appropriate use of mobile technology for informal learning and CPD. This addresses the concept of digital professionalism, which embodies ethical use and maintenance of professional boundaries when using mobile technology within healthcare environments. One participant suggested learning about safe and appropriate use was a risk management strategy to ameliorate the current circumstances:

*“But of course, that's again, I don't think - I think that's the risk but I - my philosophy is let's train people, let's have a policy, let's train people in safe, responsible mobile use” (Participant 2).*

Another participant pointed out nurses need to know their professional boundaries regarding seeking and retrieving information, and users must be able to critically reason when it is appropriate to use mobile technology for learning:

*“But as I said, nurses need to learn what they need to learn when they need to learn it. This can augment that process but again we’re not going to learn how to do open heart surgery just because we’ve got a new device that’s got it there for us. We still have to have appropriate use” (Participant 6).*

Within the theme of risk management, it became apparent that nurse representatives belonging to nursing profession organisations acknowledged there was an issue in the workplace. However, due to the volunteer nature or absence of priority to enable informal learning of CPD at point of care within these organisations, there was a lack of agency to drive change, to enable mobile learning to become a legitimate nursing function. A representative indicated:

*“I think that we could - and we're doing it at the moment, slowly, as you know, these volunteer organisations and colleges are slow-moving ships but we are trying to develop a policy, not so much about - it probably won't be specific about mobile learning” (Participant 2).*

Interviewees indicated from their comments that despite the lack of congruency about mobile technology use at point of care, they did not view themselves as responsible for solving the current paradox. Representatives discussed the issue as though it was outside the aim and scope of their professional organisation to effect change. There was no acknowledgement of the capacity of their organisations to advocate for a change in the status quo or to show leadership in the National arena relating to accessing mobile learning even though it could potentially benefit their members and patients. One participant stated:

*“But we specifically don’t have a position statement on it, it’s just something that we recognise is a minimum standard that it must be” (Participant 5).*

### Perceived use of mobile technology

From the comments by representatives of nursing profession organisations it was clear that non-use of mobile learning in nursing healthcare environments is commonplace. Statements by interviewees indicated that healthcare lags behind other industries in harnessing emerging technology and nursing is hindered by groups, within and external to the profession. Representatives provided a range of examples where other stakeholders including the medical profession were using mobile technology. Stakeholders in this context were individuals and organisations that interact with, or impact the opportunities of, participants to access or use mobile technology at point of care. For example, one participant indicated junior medical officers (JMOs) could access mobile learning at point of care:

*“Well yeah, it certainly seems to be that it’s - I see certainly - I guess, I’m getting a little bit older - see a lot new, younger - you know, the JMOs and even some of the residents coming though and they use their phones constantly and it doesn’t seem to be seen as an issue” (Participant 5).*

Participants indicated there was a need to address how patients perceive mobile learning by nurses if mobile technology is to be used at point of care. A participant indicated they perceived patients were unaccepting of nurses using mobile technology:

*“Because I think that there is perhaps a perception and as I said particularly from older people out there that we're using phones merely to communicate with our friends as opposed to actually looking up things that are useful for the conversation at hand” (Participant 3).*

Another representative commented they believed patients would be accepting if the purpose of using the technology was explained:

*“However, from a patient’s perspective, also from the perspective on a personal level, if you use it with them and you explain what you’re doing they’ll often be quite accepting of that” (Participant 4).*

Additionally, interviewees indicated there was fear of reputational damage, if nurses were accused of misuse by other stakeholders. This risk influenced whether nurses accessed mobile technology at point of care. One representative indicated:

*“I think they’ve felt - well, there’s been complaints from a patient perspective that nurses seem to be on their phones, using their phones. They see it as patient perception, that nurses in particular aren’t working, they’re using their mobile devices for personal use in the workplace rather than using it for work purposes” (Participant 4).*

Participants indicated generational cohorts of patients and co-workers behaved differently and this behaviour needed to be taken into account when using mobile technology for learning:

*“But we’ve got - it’s perception from a different generation that doesn’t see it the same way necessarily, so there needs to be education around ‘this is what’s happening with these mobile devices’ as well. Whereas, I certainly see the younger generations now - so, gen Y will often use online learning. So, not necessarily mobile technology as such but they will use Internet learning far more readily” (Participant 4).*

Furthermore, they suggested that access to mobile technology varied depending on the role of the nurse. One interviewee stated:

*“But the nurses, just generalist nurses, certainly aren’t able to use their - or are discouraged from having their phones on them when they’re with patients” (Participant 4).*

Participants believed historical circumstances contributed to the current situation where the nursing profession trails other health professions in using mobile learning. For example, one representative stated:

*“And I think that while there might be a little bit of a backlash from people who are yearning for a bygone time, the reality going forward is that this reflects well on nursing, showing that nursing is very professional, that they are engaging in and embracing technology” (Participant 6).*

### Connectivity to information

Connectivity to information was viewed by interviewees as crucial for enabling informal learning and CPD at point of care. Connectivity to information in this context includes the tangible and intangible consequences of stakeholder interactions using mobile technology for information transfer. Representatives indicated they believed it was detrimental to the work of nurses to block access to information transfer for the purpose of connecting with others, or for seeking and retrieving information via the Internet. Nurses needed to demonstrate they were professional, capable and contemporary in their role as one participant stated:

*“If in the aviation industry, if our bookings were done by paper we’d be going what’s going on here?…I think most people prefer, to have a nurse turn up with a digital device or something to be accessing information” (Participant 6).*

Statements by participants indicated Internet connectivity to undertake their clinical role was hidden. For example, one participant provided an explanation about why they perceived nurses were unable to harness mobile learning at point of care:

*“I wonder whether nurses tend to be seen as giving that hands on physical care, so they can’t pull their phone out and use it, whereas doctors if they’re consulting and so it’s all right for them to be looking at their phone and that they’re being seen to use it for work purposes” (Participant 4).*

The inability of nurses to promote their knowledge and skills hinders their access to this vital resource in the new learning age. Nurses are viewed as caring and compassionate and their high level of clinical skills that can be augmented by knowledge management through connection to the Internet, is less overt. As stated by one participant the need for access to mobile learning tools and resources to improve patient outcomes is invisible.

*“Anyway, it's actually detrimental because it's a really useful tool, these mobile devices, for our staff” (Participant 2).*

### Real-time access

Real-time access refers to whether participants have the ability to connect at the actual time to transfer information using mobile technology. Interviewees were enthusiastic about the potential of mobile learning at point of care from the perspective that information was available when required. One participant stated:

*“I mean, I've worked in nursing for a hell of a long time and I think I would have given my left arm for that type of ability to look things up then and there at the time” (Participant 3).*

Participants recognised the convenience of being able to access information as required without leaving the patient. One representative reported:

*“Because we’re busy working. We haven’t got time to be always stopping to do things. We’re busy. And the modern life is busy. And I actually think that nurses find out what they need to know when they need to know it” (Participant 6).*

Similarly, another representative revealed the belief that slow acceptance of mobile learning into healthcare environments hindered the advance of nursing practice:

*“And in health, I think technology generally in health is really underutilised and I think that we could become far more efficient with education and in improved patient care by using it more appropriately” (Participant 4).*

Comments about the inability to harness mobile learning at point of care indicated that stakeholders were missing vital information and interactions that could improve patient outcomes. One example demonstrates the broad scope of mobile learning for clinicians in practice:

*“Whereas we are looking up, we should be looking up blood results and then checking it on an app on your phone and finding out what that could be and looking at with your patient symptoms would be fantastic if nurses were doing that and I think would save a huge amount of patient deterioration and improve care” (Participant 4).*

Additionally, participants recognised the benefits for nursing students of being able to learn in real-time:

*“So, it is really that point at which they know that it’s going to be significant for them [students] and if they were to like make a note for themselves like we used to do when we were on clinical placement to go and look it up at home. Well sometimes you don’t get there, don’t do it for one reason or another you forget” (Participant 3).*

Interviewees also realised that over time learning in real-time at point of care will become more commonplace:

*“Yes, I think so. You’ve got to move with the times. I realise that, over the next decade or so, we’ve got an older patient group but my mother’s downloaded recipes off the internet. I think that’s an excuse. I think we have to move. It’s in the banking industry. Every other industry That’s just part of society. I don’t think it’s any different in nursing. I think though that nurses in the public image are a little bit caught in time, in a bit of a time capsule. And we’re not allowed to grow up” (Participant 6).*

Participants recognised that access to learning in real-time will take leadership and concerted effort by stakeholders:

*“But I think there’s still a lot of work to be done in being able to do it, I don’t think there’s a magic bullet that will make it happen but rather a sort of concerted effort over a period of time” (Participant 5).*

One representative summed up the future of mobile learning by stating:

*“Easily accessible up to date information on the device in your hand at the time you’re standing by the patient” (Participant 1).*

## Discussion

**Fig. 1:**
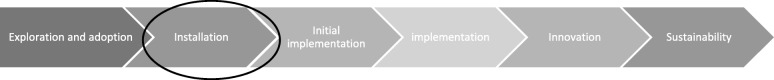
Stages of implementation (Modified from Fixsen et al. [43])

The emergent themes of risk management, perceived use of mobile technology, connectivity to information and real-time access in this research confirm Fixsen and colleagues’ [43] framework that mobile learning is stalled at the adoption point in the Stages of Implementation (Fig. 1). The four themes support the contention that nurses within nursing profession organisations are currently unwilling to lead on installing mobile learning at a systems level. This reluctance to advance access and use of mobile technology for learning within national healthcare environments then flows over to organisational and individual levels. The absence of clear direction within the Registered Nurse Standards for Practice [6] and new draft Codes of Professional Standards [44] illustrates the issue and compounds the problem of lack of governance. The leadership vacuum within and outside the nursing profession in favour of reform is perpetuating the mobile learning paradox.

Action across all four themes is necessary to enhance governance for mobile learning at point of care. As long as the identified limitations persist, nurses will be hindered in their access to mobile learning for informal learning and CPD. Additionally, nurses cannot support, guide or model digital professionalism to nursing students undertaking work integrated learning. The unwillingness of senior nurses to lead on mobile learning is a cause for concern since it is required to overcome the observed stalled implementation [3, 43]. For progress to be made, developing protocols that address the four identified themes will be required.

The release of Australia’s National Digital Health Strategy [45] and review of Registered Nurse Accreditation Standards [46] has created opportunities to remedy the current situation by establishing a governance structure within organisations that individuals can implement. Strategy 6 of the Digital Health Strategy acknowledges that Australia requires a health workforce that can confidently use digital health technologies to deliver health and care [45]. Support for change management, training, resources and clear direction are outlined. Additionally, the Australian Nursing and Midwifery Accreditation Council Consultation Papers 1 and 2 provide opportunity to feed forward information about supporting health informatics and mobile learning within the undergraduate nursing curriculum [46, 47].

Nurses are bound by National Standards and Codes which provided detailed cues about expected knowledge, skills, attitudes and behaviour of nurses. The new Registered Nurse Standards for Practice [6] and revised Codes [44] are more generic, giving organisations and individuals more autonomy to determine expectations of nursing practice [16]. However, the lack of explicit information regarding mobile technology in these documents appears to be discouraging its use in healthcare environments because nurses are not yet conversant with the new Standards and Codes and the level of autonomy they provide [48]. The research has demonstrated that senior nurses are unwilling to lead workplace change and have little enthusiasm for being involved in the change process.

Most participants did not view themselves as playing an advocacy role within their nursing profession organisation with regard to mobile learning. Those who did thought that change within professional organisations was slow because it usually relied on volunteer labour, which waxed and waned depending on individual circumstances. Volunteer ‘burnout’ led to inconsistency in progressing the aims and objectives of the nursing profession organisation. In addition, the main focus of specialty organisations is advancing specific clinical information and advocating for new platforms to convey that information is not envisaged. Finally, nurses who hold executive positions within nursing profession organisations often do not provide direct care and thus lack contemporary experience of the new ways information can be integrated into nursing practice and transferred at point of care. Thus, until there is a greater appreciation of the issues, the current lack of leadership will continue to hinder progress towards implementation [41].

It is also evident there is a lack of consistency in knowledge, attitudes and behaviour within the nursing profession regarding the use of mobile technology. Resistance to changing workflows [36] owing to inadequate educational preparation and fear of inappropriate use of mobile technology was reported [3, 20]. Representatives provided examples where inappropriate behaviour resulted in the ‘banning’ of mobile technology at the workplace and anecdotal evidence of previous inappropriate behaviour of health professionals [20, 49, 50] has shaped the current situation. Interviewees justified the inequity of access by claiming adverse media attention was responsible [20, 51, 52]. Participants mentioned cyberloafing and unprofessional behaviour such as using social media while at the workplace contributed to the inability to use mobile technology [53, 54]. All representatives narrated stories of inappropriate behaviour by nurses while admitting they had not witnessed it themselves.

Direct care nurses were unable to access mobile technology, whereas nurses in other roles were allowed to carry a mobile device. This shifting access to mobile technology perpetuates confusion between leisure and learning and will only be ameliorated when mobile learning becomes a legitimate nursing function [55]. Continuance of the lack of governance that supports the mobile learning paradox will impede implementation of mobile learning at point of care. Since further innovation in mobile technology is predicted [56, 57], the current mobile learning gap will continue to widen if the status quo remains unchallenged.

Access to learning resources within healthcare environments is an important imperative. Currently, however, seeking and retrieving relevant information in real-time by nurses is hidden. While nurses are viewed by the public as caring and compassionate individuals, their advanced critical thinking and capacity for managing complex nursing care more covert and less recognised [58]. Therefore, clinical skill enhancement by accessing information in real-time is underappreciated by organisations and nurses. The difficulty in demonstrating the value of access to information transfer in real-time is also arresting progress towards the implementation phase.

As highly skilled clinicians, nurses are constantly analysing and altering their planned schedule of care as new information or events require [36]. Constant interruptions to established workflows require critical thinking and an ability to be flexible. As interruptions to workflow increase, the fragmentation of nursing care creates the need for workarounds. Nurses modify the way they think and behave when practices no longer work as intended, become redundant or opportunities occur to incorporate new work practices that benefit workflow. This adaptation process includes recognising the new intervention’s benefits and investing in learning about the new process to enable integration into routine work patterns. Sustaining change occurs when the benefits outweigh non-use [59]. This process is being attenuated with regard to mobile technology and mobile learning, however. From the interviews, nurse leaders appear to absolve themselves of responsibility for advocating within the profession to advance nursing practice. Nurses continue to support a historically hierarchical system that justifies their lack of inclusion in decision-making and are consequently unable to articulate the importance of mobile learning for enabling informal learning and CPD [58]. This apparent inability to communicate the value of access to mobile technology is hindering nurses’ capacity to demonstrate how mobile learning improves workflow, promotes continuity of care and potentially improves patient outcomes. It also prevents the modelling of digital professionalism to undergraduate nurses perpetuating the status quo. The current deficiency in the capacity of nurses to influence the direction of mobile learning policy at system and organisation levels further marginalises them within the registered health professions [16, 60].

The casualties of this failure to embrace the mobile learning era include a range of stakeholders. An inability to engage in mobile learning at point of care is a lost opportunity for experienced nurses to lead learning in real-time at the workplace. Being able to legitimately access information at the bedside has the potential to build capacity with other health professionals, students and patients [61]. Moreover, accessing mobile technology at point of care could strengthen the nurse-patient relationship by increasing mutuality of understanding [1], enable continuity of care and reduce time away from the patient. Nurse supervisors could capitalise on real-time learning moments by supporting students at point of care by using mobile learning when it is safe and appropriate to do so. Currently, nurses support students in practice because they believe ‘it is the right thing to do’ [48]. However, although they understand the risks, challenges and benefits, they do not advocate for access to mobile learning to support this activity. This unwillingness to lobby for access to learning resources confirms the noted absence of agency by nurses to contemporise their nursing practice by maximising opportunities for informal learning, CPD [62] and teaching students undertaking work integrated learning.

The inclusion of mobile learning early in the nursing curriculum in the classroom will enable modelling of digital professionalism to occur prior to undertaking work integrated learning [24]. Consistency between learning on campus and being able to continue to use mobile technology during work integrated learning will promote safe and appropriate use by the next generation of nurses. The ADHA Digital Health Strategy [45] acknowledges the need for preparation of the nursing health workforce to become digitally literate. As the nursing workforce is the largest of the registered health professions it is imperative that resources are channelled to upskill the current workforce [63]. It is also imperative that nursing profession organisations recognise that knowledge management relies on connectivity to information and that they have a responsibility to advocate for appropriate governance of mobile learning at point of care for the benefit of all stakeholders. Only when there is greater equity of access to mobile technology will nurses be fully able to participate in informal learning, CPD, and training nursing students in digital professionalism and thus to deliver contemporary nursing practice in real-time.

### Impact statement

Lack of governance guiding the use of mobile technology at point of care at a systems level negatively impacts the ability of nurses to legitimately incorporate mobile learning into their nursing practice. The current ‘mobile learning paradox’ needs to be resolved from within the profession of nursing and healthcare organisations. Perpetuation of the mobile learning paradox has implications for the profession internationally, where governance structures regarding access and use of mobile technology in healthcare environments has not been addressed.

### Limitations

Limitations of this study include timing of interviews, which due to the short recruitment period took place during December 2016 – January 2017. Recruitment in the lead up to the Christmas period may have reduced opportunity as potential participants may have organised annual leave during the Australian summer, were required to complete work by the end of the year or work during the traditional holiday shut-down period may not have responded, whereas they have done so if recruitment occurred during another time period. Recruitment ceased when the Health Informatics Society of Australia, Nursing Informatics Australia and Australian College of Nursing released the joint draft position statement on nursing informatics in February 2017, as this could have changed perspectives of future interviewees by raising awareness of the topic.

### Strengths

Although recruitment numbers were low, participants were senior nurses, who during their careers had experienced clinical, administration, education and research within the nursing profession. This wealth of knowledge was demonstrated through interview. Timing of the interviews was a limitation, and also a strength. This study was undertaken before the draft position statement on nursing informatics was released, providing baseline understanding of the field that can provide direction for further research.

### Future directions

The nursing profession is the largest of the registered health profession. As such, this profession is in a strong position to lead mobile learning at point of care. However, this ascendancy will only be accomplished when nurses marshal their mobile learning agency by taking responsibility for leadership within healthcare environments.

There is an opportunity to achieve this aim by embracing the ADHA National Digital Health Strategy [64] and demanding the profession of nursing is included in decision-making at a systems and organisation level. Involving nurses in systems design and creating positive and supportive environments is instrumental to sustainability of the health workforce [65]. Further research into safe and appropriate use of mobile learning by trialling its use needs investigation. The inclusion of digital professionalism early within the undergraduate nursing curriculum is necessary, as is the educational preparation of undergraduate nurses and nurses currently employed within healthcare settings. It is imperative that nurses develop requisite skills to seamlessly undertake patient care and to guide and support students in using mobile technology for learning at point of care.

Further research into mobile learning at point of care is necessary to ensure standards, guidelines and codes of conduct reflect safe and appropriate use. Usability trials to evaluate quality and safety issues may assist with providing evidence to guide risk management for implementation of mobile learning at point of care. This research will also provide rich data to guide undergraduate nursing curriculum development. Gaining the patient perspective regarding nurses using mobile learning will be beneficial to all stakeholders. Findings can be used to guide patient education about the implementation of mobile learning and be used to guide deployment of mobile learning in health care environments.

## Conclusions

There is a gap in the governance of mobile technology for learning by nursing profession organisations. At systems and organisation levels, there is a lack of leadership providing direction for the professional conduct of nurses, which is expressed as the inability for nurses to implement mobile technology for learning as a legitimate nursing function. This shortage of support stalls the capacity for individual nurses to implement and model digital professionalism at point of care. Additionally, there is a deficiency of agency within the nursing profession and healthcare organisations that further hinders the installation or deployment of mobile learning at an individual level within healthcare environments.

Through their narratives participants indicated that an absence of governance within nursing organisations is perpetuated by a lack of inclusion in decision-making at a systems level. It is evident from this study that there is insufficient agency by nurses in leadership positions to influence the installation of mobile technology for informal learning and CPD at point of care in healthcare environments. However, inclusion of nurses in healthcare decision-making at a systems level coupled with promoting digital professionalism within organisations and higher education institutions will foster a more inclusive culture that will contribute to improving patient outcomes.

The installation of digital technology for mobile learning to enable informal learning and CPD to be undertaken at point of care challenges traditional work patterns. There is a lack of leadership by nurses within professional organisations to advance governance of digital professionalism that needs to be ameliorated. Empowerment of members within nursing profession organisations will support mobile learning to become a legitimate nursing function.

## Abbreviations

ADHA: Australian Digital Health Agency
CoNNMO: Coalition of National Nursing and Midwifery Organisations
CPD: Continuing Professional Development
